# MiD51 Is Important for Maintaining Mitochondrial Health in Pancreatic Islet and MIN6 Cells

**DOI:** 10.3389/fendo.2020.00232

**Published:** 2020-04-28

**Authors:** Julia Schultz, Jeanette Warkus, Carmen Wolke, Rica Waterstradt, Simone Baltrusch

**Affiliations:** ^1^Institute of Medical Biochemistry and Molecular Biology, University Medicine Rostock, Rostock, Germany; ^2^Institute of Medical Biochemistry and Molecular Biology, University Medicine Greifswald, Greifswald, Germany; ^3^Department Life, Light & Matter, University of Rostock, Rostock, Germany

**Keywords:** MiD51, mitochondrial dynamics, mitochondrial fission, mitophagy, pancreatic beta cells

## Abstract

**Background:** Mitochondrial dynamics are important for glucose-stimulated insulin secretion in pancreatic beta cells. The mitochondrial elongation factor MiD51 has been proposed to act as an anchor that recruits Drp1 from the cytosol to the outer mitochondrial membrane. Whether MiD51 promotes mitochondrial fusion by inactivation of Drp1 is a controversial issue. Since both the underlying mechanism and the effects on mitochondrial function remain unknown, this study was conducted to investigate the role of MiD51 in beta cells.

**Methods:** Overexpression and downregulation of MiD51 in mouse insulinoma 6 (MIN6) and mouse islet cells was achieved using the pcDNA expression vector and specific siRNA, respectively. Expression of genes regulating mitochondrial dynamics and autophagy was analyzed by quantitative Real-Time PCR, glucose-stimulated insulin secretion by ELISA, and cellular oxygen consumption rate by optode sensor technology. Mitochondrial membrane potential and morphology were visualized after TMRE and MitoTracker Green staining, respectively. Immunofluorescence analyses were examined by confocal microscopy.

**Results:** MiD51 is expressed in insulin-positive mouse and human pancreatic islet and MIN6 cells. Overexpression of MiD51 resulted in mitochondrial fragmentation and cluster formation in MIN6 cells. Mitochondrial membrane potential, glucose-induced oxygen consumption rate and glucose-stimulated insulin secretion were reduced in MIN6 cells with high MiD51 expression. LC3 expression remained unchanged. Downregulation of MiD51 resulted in inhomogeneity of the mitochondrial network in MIN6 cells with hyperelongated and fragmented mitochondria. Mitochondrial membrane potential, maximal and glucose-induced oxygen consumption rate and insulin secretion were diminished in MIN6 cells with low MiD51 expression. Furthermore, reduced Mfn2 and Parkin expression was observed. Based on MiD51 overexpression and downregulation, changes in the mitochondrial network structure similar to those in MIN6 cells were also observed in mouse islet cells.

**Conclusion:** We have demonstrated that MiD51 plays a pivotal role in regulating mitochondrial function and hence insulin secretion in MIN6 cells. We propose that this anchor protein of Drp1 is important to maintain a homogeneous mitochondrial network and to avoid morphologies such as hyperelongation and clustering which are inaccessible for degradation by autophagy. Assuming that insulin granule degradation frequently suppresses autophagy in beta cells, MiD51 could be a key element maintaining mitochondrial health.

## Introduction

Mitochondria are highly dynamic organelles that constantly move in living cells and undergo fusion and fission events (1–4). Continuous adaptation of the mitochondrial network morphology is pivotal to maintain the physiological cell response. Dysfunction of mitochondrial dynamics is proposed to contribute to several diseases (5–8) and has also been shown to be involved in the pathogenesis of type 2 diabetes mellitus (9–13). Fission is vital to recover mitochondrial health by counteracting organelle hyperfusion and by opening up damaged mitochondrial areas to degradation (4, 14–16).

The recruitment of dynamin-related protein 1 (Drp1) from the cytoplasm to the outer mitochondrial membrane and subsequent oligomerization is the essential initial step for fission (17–21). Unlike dynamin, Drp1 does not contain a transmembrane domain and thus relies on anchor proteins to constrict and eventually split a mitochondrion (22). In mammalian cells different receptors located in the outer mitochondrial membrane—in particular fission protein 1 (Fis1) and mitochondrial fission factor (Mff)—have been proposed to act in Drp1 recruitment (22). However, the so-called mitochondrial elongation factors MiD49 and MiD51 (17–21) are also likely to be involved in the process. Several studies have postulated that MiD51 interacts with both Drp1 and Mff, thereby serving as an adaptor to form a trimeric Drp1-MiD-Mff complex (23–28). It has been suggested that MiD51 acts as a negative regulator of mitochondrial fission by suppressing Drp1 function and supports mitochondrial elongation (22, 29, 30). Another recent publication describes MiD51 as a positive regulator of fission and points out that dimerization of the protein is required for mitochondrial dynamics regulation (31). Investigations have not yet been conducted to establish whether MiD51 is expressed in pancreatic beta cells and impacts mitochondrial quality control.

While fission is the integral driver of the continuous mitochondrial network dynamics that ensure metabolic activity, both fusion, and mitophagy are additionally important to achieve the necessary balance. Fusion of the outer and inner mitochondrial membrane is controlled by the GTPases mitofusin 1 and 2 (Mfn1/Mfn2) and optic atrophy 1 (OPA1), respectively, and high expression and activity of these proteins cause elongation (14, 32, 33). Mitophagy is a selective way of disposing of defective mitochondria by autophagy (34). This process is initiated by accumulation of the (PTEN)-induced putative kinase (PINK1) and the ubiquitin ligase Parkin on the outer mitochondrial membrane. Loss of any of these proteins results in failure of selective removal of damaged mitochondria (15, 35, 36). However, the final degradation of mitochondria depends on the formation of autophagosomes, a process that is mainly conducted by the autophagosome marker microtubule-associated protein 1 (MAP1) light chain 3 (LC3) (37).

Because mitochondrial metabolism plays a pivotal role in glucose-stimulated insulin secretion, any changes will result in beta cell overload (38–42). Specifically, accumulation of damaged and dysfunctional mitochondria in beta cells is associated with oxidative stress, loss of respiratory control, and apoptosis (39, 43). In previous work we demonstrated that expression of both Fis1 and Drp1 have to be adapted precisely to maintain pancreatic beta cell function (44, 45). However, the relationship between the two proteins is still unclear in mammalian cells. To elucidate this question, the present study set out to overexpress and downregulate MiD51 in pancreatic beta cells to gain further insight into mitochondrial dynamics and the impact of this mitochondrial adaptor protein on beta cell function.

## Materials and Methods

### Cell Culture and Primary Pancreatic Islets

Mouse insulinoma 6 (MIN6) cells were cultured in DMEM media containing 25 mmol/l glucose supplemented with 10% fetal bovine serum and 5% penicillin/streptomycin in a humidified atmosphere at 37°C and 5% CO_2_. Mouse pancreatic islets were isolated from 12-week-old male NMRI mice by collagenase P (Roche Diagnostics, Mannheim, Germany) digestion and Ficoll gradient purification (Ficoll PM 400; Sigma, Seelze, Germany). This procedure was conducted in accordance with the German Animal Welfare Act 2006 (last amended 2014) and was approved by the State Department of Agriculture, Food Safety and Fisheries, Mecklenburg-Vorpommern (LALLF M-V). Mice were housed at the central animal care facility of the Medical Faculty, University of Rostock, receiving conventional rodent chow and water *ad libitum*. Human adult pancreatic islets were donated from biopsies performed during pancreatic surgery, as approved by the ethics committee of University Medicine Greifswald (BB 050/13). Primary islets were seeded and expanded on x-well Tissue Culture Chambers for confocal microscopy (Sarstedt, Nümbrecht, Germany), and cultured in RPMI 1640 media containing 11 mmol/l glucose supplemented with 10% fetal bovine serum and 5% penicillin/streptomycin, 1 mmol/l sodium pyruvate, 10 mmol/l HEPES, and 200 mmol/l glutamine in a humidified atmosphere at 37°C and 5% CO_2_.

### Overexpression and Silencing of MiD51 in Pancreatic Beta Cells

Overexpression of MiD51 in MIN6 cells and primary mouse islet cells was achieved using the expression vector pcDNA3.1. Cells were transfected with pcDNA3.1(–)MiD51 4xMycHisx6 vector (Plasmid #44598, Addgene, Cambridge, MA) for 48 h using Effectene Transfection Reagent (Qiagen, Hilden, Germany) according to the manufacturer's instructions. Empty vector transfection served as control. MiD51 was downregulated in MIN6 cells and primary mouse islet cells by specific siRNA (Thermo Fisher, Waltham, MA, USA). A negative control siRNA (Thermo Fisher) was used for comparison. Cells were incubated for 48 h using Interferin transfection reagent (Polyplus, Illkirch-Graffenstadey, France).

### Quantitative Real-Time-PCR

Total RNA samples were prepared using the RNeasy total RNA isolation kit (Qiagen, Hilden, Germany) and quantified using a spectrophotometer (ND-2000, PeqLab, Erlangen, Germany). Reverse Transcription was performed using the Maxima^®^ First Strand cDNA Synthesis Kit (Fermentas, St. Leon-Rot, Germany). Gene expression was analyzed using the TaqMan Universal PCR Master Mix and the following TaqMan gene expression assays (Applied Biosystems, Carlsbad, CA, USA): Mm_00724569_m1 and Hs_01007730_g1 for MiD51, Mm_00550827_m1 for PINK1, Mm_00450187_m1 for Parkin, Mm_04225236_g1 for LC3, Mm_01255785_m1 for Mfn2, Mm_01342903_m1 for Drp1 and Mm_01288627_g1 for VDAC1. RT-PCR reactions were performed in triplicates using the 7900HT Real-Time PCR System (Applied Biosystems, Carlsbad, CA, USA). The relative expression levels were calculated with the comparative (2^−^ΔΔCt) method and normalized to ß-actin (Mm_02619580_g1 and Hs_01060665_g1) gene expression.

### Western Blot Analyses

Cells were extracted using radioimmunoprecipitation assay (RIPA) lysis buffer (50 mmol/l Tris-HCl pH 7.4, 150 mmol/l NaCl, 1% Triton X-100, 1% sodium deoxycholate, 0.1% SDS, 1 mmol/l EDTA, protease inhibitors) and centrifuged for 15 min at 12.000 g. 30 μg protein were separated by SDS-PAGE and blotted onto Roti^®^Fluoro PVDF membrane (Roth, Karlsruhe, Germany). Membranes were incubated for 1 h at room temperature with the following primary antibodies: anti-c-Myc (1:1,000) and anti-GAPDH (1:1,000) (Santa-Cruz Biotechnology, Santa Cruz, CA, USA) or anti-MiD51 (1:1,000) (Proteintech, Rosemont, IL, USA) and anti-beta-actin (1:200) (Santa Cruz). Immunoreactive bands were visualized using the following fluorescence-labeled secondary antibodies: IRDye 680 CW, IRDye 800 CW and analyzed via the Odyssey imaging system. Densitometry measurements of bands were performed using the Odyssey infrared imaging system (LI-COR, Lincoln, NE, USA).

### Immunohistochemistry

For immunohistochemistry analyses cells were seeded on x-well Tissue Culture Chambers (Sarstedt, Nümbrecht, Germany) and transfected for 48 h. Cells were fixed with 4% formaldehyde for 15 min and permeabilized with 0.2% Tween20 for 5 min in phosphate-buffered saline. Cells were stained for 1 h with the primary antibodies: anti-MiD51 (1:100) (Proteintech), anti-Tom20 (1:100) (Abcam, Cambridge, UK), anti-insulin (1:100) (Abcam) and anti-LC3 (1:100) (Sigma). Cy5 or FITC-coupled secondary antibodies (1:250) were used for visualization (Molecular Probes Invitrogen, Darmstadt, Germany). Cells were mounted and counterstained using Roti^®^-Mount FluorCare DAPI (Roth, Karlsruhe, Germany) and analyzed using a Fluoview FV10i confocal microscope (Olympus, Hamburg, Germany).

### Mitochondrial Morphology Staining

5 × 10^5^ MIN6 cells were seeded and grown on glass-bottom dishes (MatTak Corporation, Ashland, MA, USA) and stained with 20 nmol/l MitoTracker^®^ Green FM (Molecular Probes Invitrogen) for 30 min at 37°C. Mitochondrial morphology was analyzed using a Fluoview FV10i confocal microscope (Olympus, Hamburg, Germany).

### Quantification of Mitochondrial Morphology

The mitochondrial network structure visualized by MitoTracker^®^ Green or Tom20 immunofluorescence staining was investigated using Imaris software (Oxford Instruments/Bitplane, Zurich, Switzerland). Mitochondria were automatically detected from 3D images by generating a surface on the fluorescence intensity channel after background subtraction and the volume for each detected object (mitochondrion) per cell was quantified. Finally, the geometric mean and the coefficient of variation of the mitochondrial volume were calculated using the Prism analysis program (GraphPad Inc., San Diego, CA, USA). An additional count was made of large objects >30 times the mean volume, and these were defined as mitochondrial clusters.

### Measurement and Quantification of Mitochondrial Membrane Potential

5 × 10^5^ MIN6 cells were seeded and grown on glass-bottom dishes (MatTak Corporation, Ashland, MA, USA). Staining was performed using 6.2 nmol/l tetramethylrhodamine ethyl ester perchlorate (TMRE, Molecular Probes Invitrogen Detection Technologies, Eugene, OR, USA) for 30 min at 37°C. Cells were analyzed using a Fluoview FV10i confocal microscope (Olympus, Hamburg, Germany) and the mean mitochondrial intensity after background correction from z maximum intensity projection images was calculated per cell (region of interest) using Fluoview software (Olympus).

### Glucose-Stimulated Insulin Secretion

MIN6 and mouse islet cells were incubated for 1 h in bicarbonate-buffered Krebs-Ringer solution without glucose, supplemented with 0.1% albumin. Subsequently, cells were incubated for 1 h in Krebs-Ringer solution containing 5.5 and 25 mmol/l glucose. Finally, the incubation buffer from each well was collected and gently centrifuged to remove detached cells. Secreted insulin in the supernatant and insulin content was measured in homogenized scraped cells by ELISA (Mercodia, Uppsala, Sweden). Protein content was analyzed by Bradford protein measurement.

### Oxygen Consumption Rate

Oxygen consumption was measured 24 h after transfection using an optode sensor technology-based system (Unisense, Aarhus, Denmark). 2 × 10^6^ MIN6 cells were incubated for 1 h in bicarbonate-buffered Krebs-Ringer solution without glucose. Subsequently, cells were incubated for 1 h in Krebs-Ringer solution containing 25 mmol/l glucose and stimulated respiration was measured over 10 min. Thereafter, 1 μM oligomycin was added and the proton leak was determined for the next 10 min. Finally, 5 μM FCCP was added and maximal respiration was measured for the last 10 min.

### Cell Viability Assay

AlamarBlue^®^ cell viability reagent (Thermo Fisher Scientific, Waltham, MA, USA) was used according to the manufacturer's instructions. The resulting fluorescence was analyzed at 530 nm with the VICTOR3™ Multilabel Counter (Perkin Elmer, Waltham, MA, USA).

### Statistical Analysis

Data are expressed as mean ± SEM. Statistical analyses were performed by unpaired Student's *t* test or ANOVA followed by Bonferroni's test using the Prism analysis program (GraphPad Inc.). Statistical significance is expressed as ^*^p < 0.05, ^**^p < 0.01, ^***^p < 0.001.

## Results

### Expression of MiD51 in Beta Cells and Pancreatic Islets

**Figure 1:**
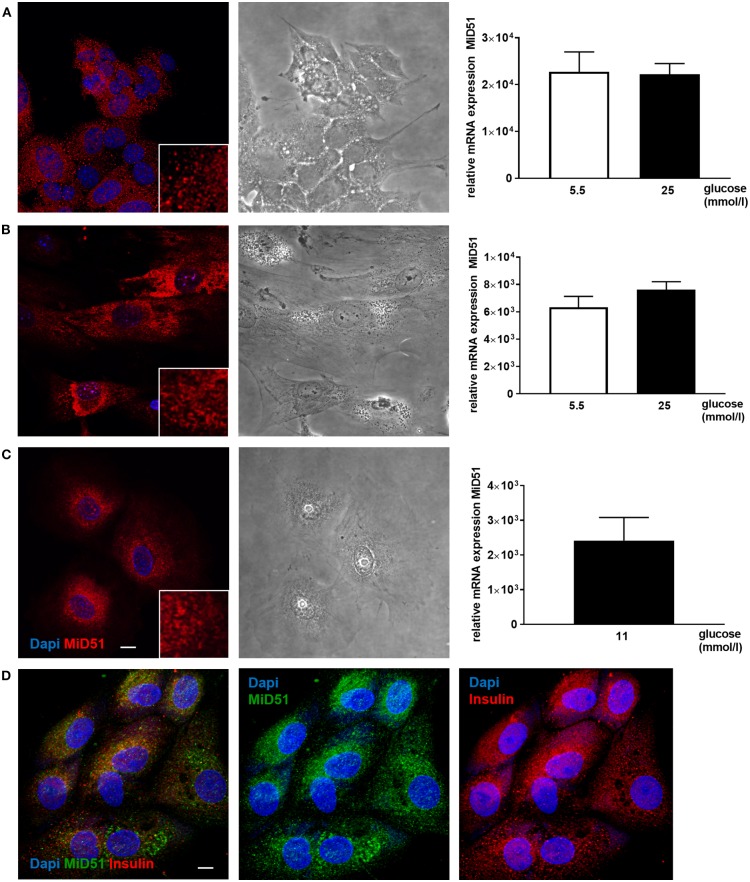
MiD51 expression in MIN6 and primary beta cells. Endogenous MiD51 protein expression is demonstrated in MIN6 (**A**, left), primary mouse islet (**B**, left) and primary human islet (**C**, left) cells with by immunofluorescence. In addition, staining of primary human islet cells with insulin and MiD51 antibodies is shown **(D)**. Representative images from three independent experiments are shown. Scale bar: 10 μm. Endogenous MiD51 gene expression is shown in MIN6 (**A**, right) and primary mouse islet (**B**, right) cells after incubation with 5.5 (white bars) and 25 mmol/l glucose (black bars), and in human islet cells after incubation with 11 mmol/l glucose (black bar) (**C**, right) for 48 h.

MiD51 expression was demonstrated in MIN6 cells and in primary mouse and adult human islets (Figure 1). The mRNA expression level of MiD51 was higher in the clonal insulin-secreting MIN6 cells (Figure 1A) than in primary mouse and human islets (Figures 1B,C); this finding was independent of glucose concentration. Immunofluorescence staining and subsequent confocal microscopy confirmed MiD51 protein expression in MIN6 cells (Figure 1A), primary mouse islet cells (Figure 1B) and human islet cells (Figure 1C) that were insulin- positive (Figure 1D).

### Enhanced and Reduced Gene and Protein Expression of MiD51 in MIN6 Cells

**Figure 2:**
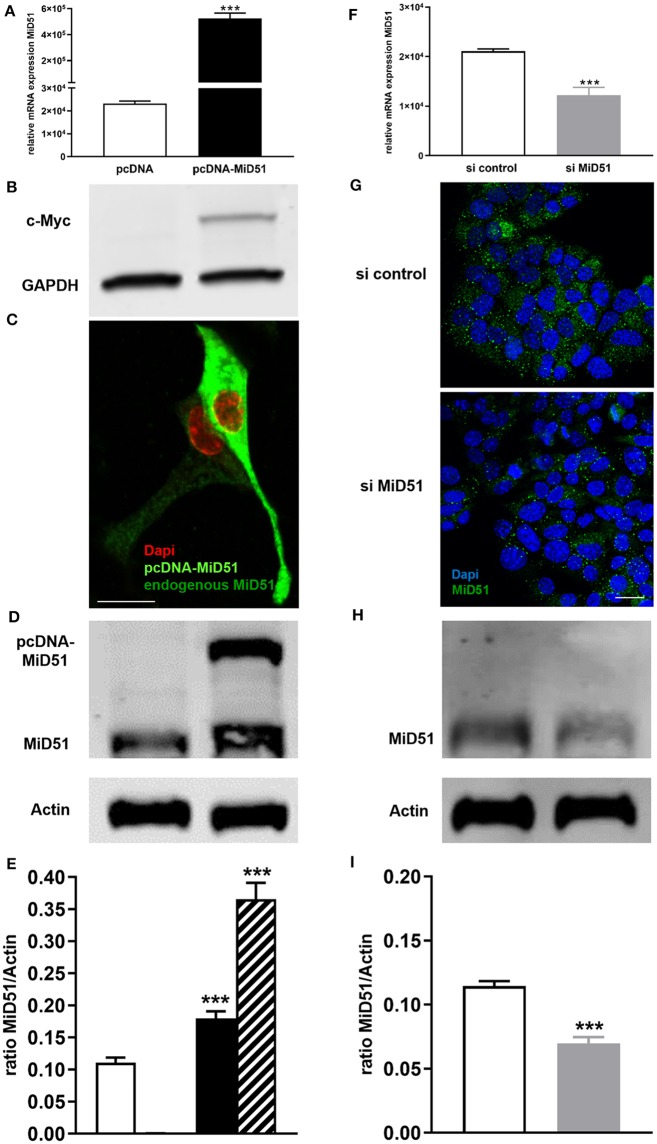
Overexpression and downregulation of MiD51 in MIN6 cells. Gene expression **(A,F)** of MiD51 in pcDNA-MiD51 transfected (**A**, black bar) and si MiD51 transfected (**F**, gray bar) compared with corresponding control transfected (white bars) MIN6 cells. Data are expressed as mean ± SEM from six independent experiments; ^***^p < 0.001 (Student's *t*-test). MiD51 protein overexpression was analyzed by western blotting using an antibody against the c-Myc tag **(B)**. Immunofluorescence analyses were performed with an antibody against MiD51 **(C,G)**. Representative images from six independent experiments are shown. Scale bar: 10 μm **(C,G)**. Protein expression **(D–I)** of MiD51 in pcDNA-MiD51 transfected (**D,E**, black and striped bar) and si MiD51 transfected (**H, I**, gray bar) compared with corresponding control transfected (white bars) MIN6 cells. Representative western blots are shown **(D,I)**. Note that the size of overexpressed MiD51 is increased due to the c-Myc tag (**B,D,E**, striped bar). Data **(D,H)** are expressed as mean ± SEM from four independent experiments; ^***^p < 0.001 (Student's *t*-test).

MiD51 overexpression was confirmed at the gene (Figure 2A) and protein (Figures 2B,D,E) level. Immunofluorescence analyses additionally demonstrated the significant difference between endogenous and enhanced levels of MiD51 in MIN6 cells (Figure 2C). Furthermore, significant MiD51 downregulation was demonstrated at the gene (Figure 2F) and protein level, both by immunofluorescence staining (Figure 2G) and western blot analyses (Figures 2H,I) compared with negative control transfected cells.

### Overexpression and Downregulation of MiD51 Results in Mitochondrial Network Inhomogeneity in MIN6 and Mouse Islet Cells

**Figure 3:**
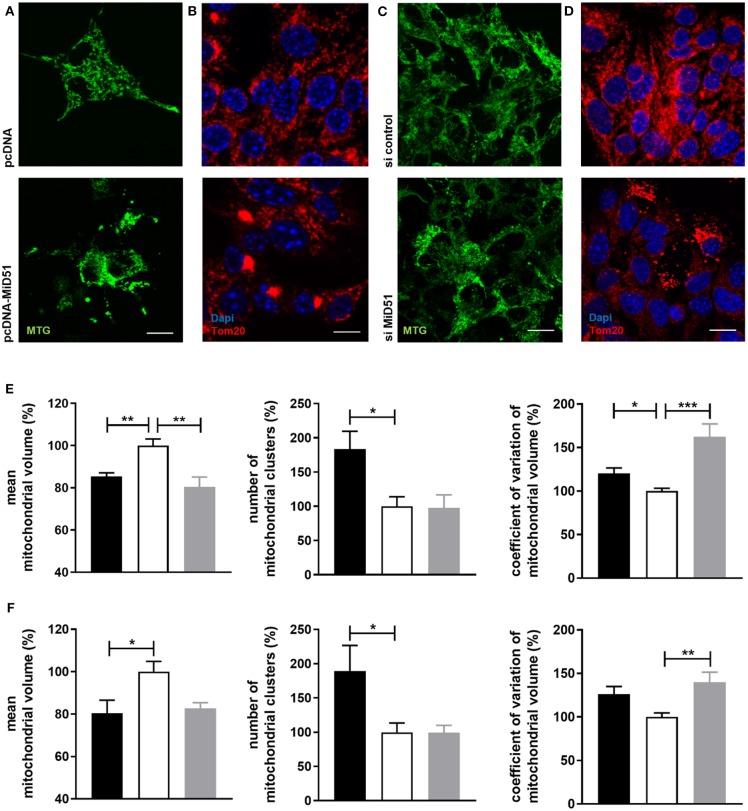
Mitochondrial morphology after overexpression and downregulation of MiD51 in MIN6 cells. MIN6 cells (transfected as indicated) were analyzed by confocal microscopy after staining with MitoTracker^®^ Green **(A,C)** or immunofluorescence staining with Tom20 **(B,D)**. Representative images from three independent experiments each are shown. Scale bar: 10 μm. The mitochondrial structure visualized by MitoTracker^®^ Green staining **(E)** or Tom20 immunofluorescence staining **(F)** of at least 20 cells per experiment was automatically analyzed and the mean mitochondrial volume, the number of mitochondrial clusters and the coefficient of variation of the mitochondrial volume were calculated. Data are expressed as mean ± SEM in % of controls (white bar) for pcDNA-MiD51 transfected (black bars) and si MiD51 transfected (gray bars) cells. ^*^p < 0.05, ^**^p < 0.01, ^**^p < 0.001 (ANOVA/Bonferroni's test).

**Figure 4:**
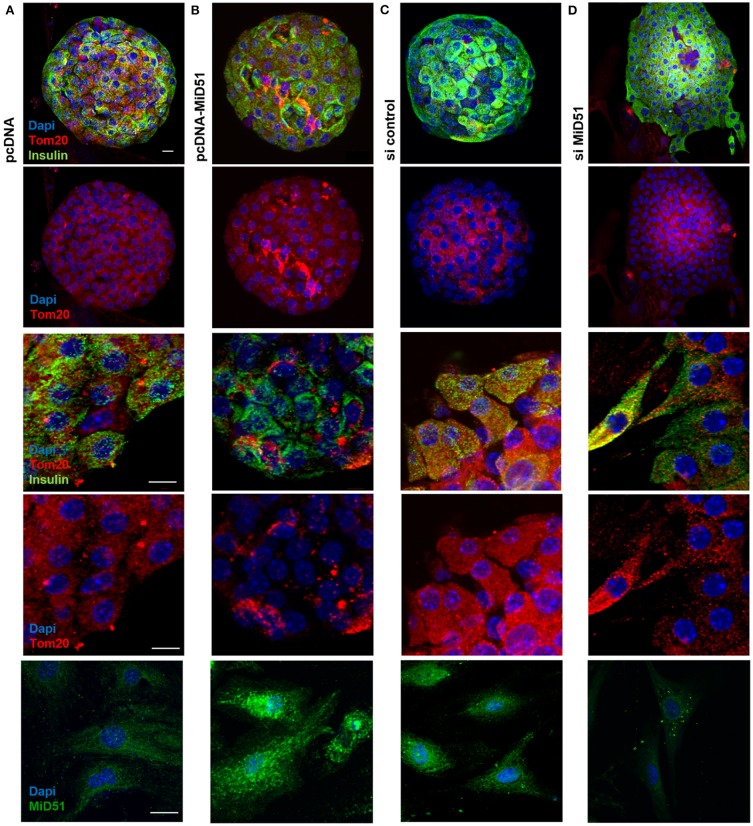
Mitochondrial morphology after overexpression and downregulation of MiD51 in primary mouse islet cells. Primary mouse islets were cultured on x-well Tissue Culture Chambers and transfected with pcDNA **(A)**, pcDNA-MiD51 **(B)**, si control **(C)**, or si MiD51 **(D)**. Finally, cells were analyzed by confocal microscopy after immunofluorescence staining with Tom20 and insulin antibodies or MiD51 antibody (bottom). Changes in mitochondrial morphology are best detectable in transfected outspread mouse islets (middle), but are also visible in whole islets (top). Representative images from three independent experiments each are shown. Scale bar: 10 μm.

Control transfected MIN6 cells (Figures 3A,B **top**) displayed homogeneous distribution of mitochondria, whereas clustered as well as fragmented mitochondria were present in MiD51-overexpressing MIN6 cells (Figures 3A,B **bottom**). In MIN6 cells with reduced MiD51 expression (Figures 3C,D **bottom**) hyperelongated and fragmented mitochondria were detectable compared with control siRNA transfected cells (Figures 3C,D **top**). In addition, the mitochondrial network was described by volume analysis (Figures 3E,F). Both, overexpression and downregulation of MiD51 resulted in a significantly higher fragmentation rate (reduced mean mitochondrial volume, left). However, whereas overexpression evoked mitochondrial cluster formation (middle), downregulation caused significantly greater network inhomogeneity (higher coefficient of variation, right) compared with control cells. Altogether comparable results were observed by mitochondrial detection via MitoTracker^®^ Green staining of living MIN6 cells (Figures 3A,C,E) and Tom20 immunofluorescence (Figures 3B,D,F) staining of fixed MIN6 cells. Similar findings were observed by investigations of primary mouse islets cultured on x-well Tissue Culture Chambers at the single (insulin-positive) cell level. Mitochondrial cluster formation was detectable after MiD51 overexpression (Figure 4B) compared with control cells (Figures 4A,C) and MiD51 downregulation mainly resulted in fragmentation (Figure 4D). MiD51 expression calculated from immunofluorescence images was increased by 48 ± 17 % after overexpression and reduced by 35 ± 19% after downregulation.

### Overexpression and Downregulation of MiD51 Changes Mitochondrial Membrane Potential and Oxygen Consumption in MIN6 Cells

**Figure 5:**
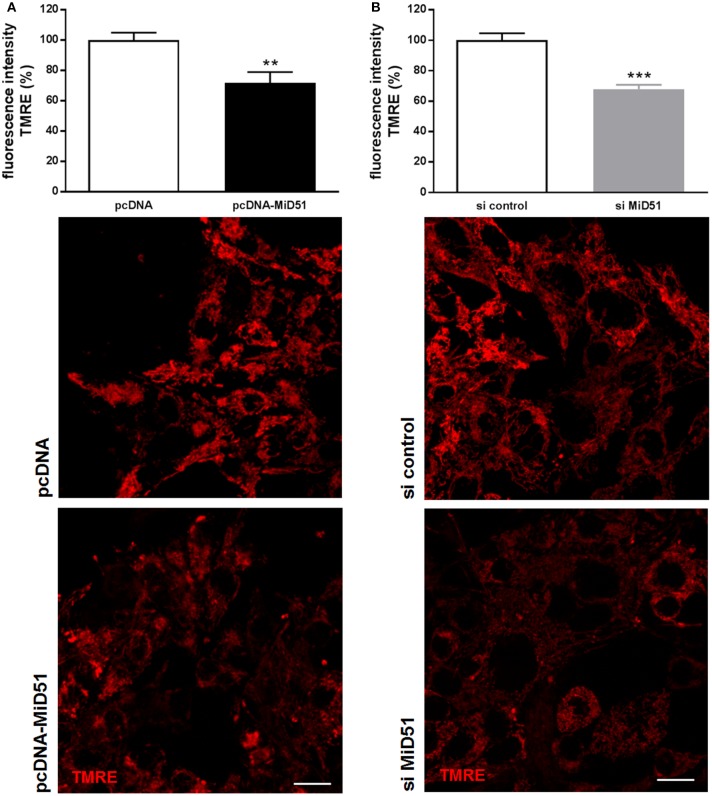
Reduced mitochondrial membrane potential in MIN6 cells after overexpression and downregulation of MiD51. Mitochondrial membrane potential was investigated in MIN6 cells using TMRE staining after transfection with **(A)** pcDNA (white bar) or pcDNA-MiD51 (black bar) and **(B)** si control (white bar) or si MiD51 (gray bar). Data are expressed as mean ± SEM from six independent experiments; ^**^p < 0.01, ^***^p < 0.001 (Student's *t*-test). Representative confocal microscopy images are shown in addition. Scale bar: 10 μm.

**Figure 6:**
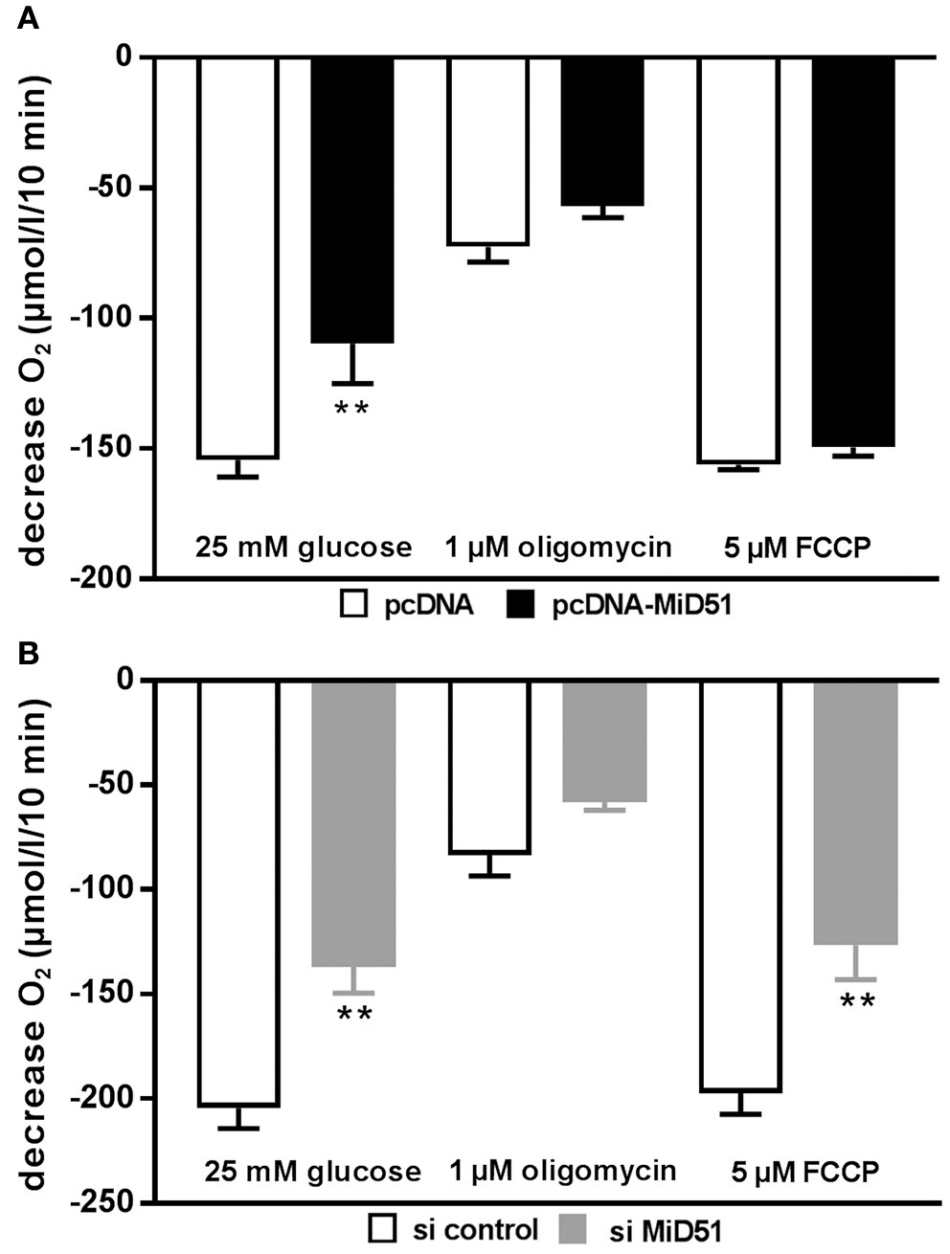
Overexpression and downregulation of MiD51 changes the oxygen consumption rate in MIN6 cells. Oxygen consumption was measured under the experimental conditions indicated in MIN6 cells after transfection with **(A)** pcDNA (white bars) or pcDNA-MiD51 (black bars) and **(B)** si control (white bars) or si MiD51 (gray bars). Data are expressed as mean ± SEM from six to eight independent experiments; ^**^p < 0.01 (ANOVA/Bonferroni's test).

Mitochondrial membrane potential was significantly reduced by 28% in MIN6 cells overexpressing MiD51 compared with control transfected cells (Figure 5A). Mitochondrial membrane potential in MIN6 cells with downregulated MiD51 expression was significantly reduced by 33% (Figure 5B). Oxygen consumption rates were also changed accordingly (Figure 6). MiD51-overexpressing MIN6 cells showed significantly lower respiration after stimulation with 25 mmol/l glucose and the proton leak after addition of 1 μM oligomycin was slightly reduced (Figure 6A). Maximal respiration after uncoupling with 5 μM FCCP was unchanged (Figure 6A). However, MIN6 cells with reduced MiD51 expression exhibited significantly lower respiration both after stimulation with 25 mmol/l glucose and after uncoupling with 5 μM FCCP (Figure 6B). The proton leak after addition of 1 μM oligomycin was again slightly reduced (Figure 6B).

### Overexpression of MiD51 Reduces Glucose-Stimulated Insulin Secretion in MIN6 and Mouse Islet Cells

**Figure 7:**
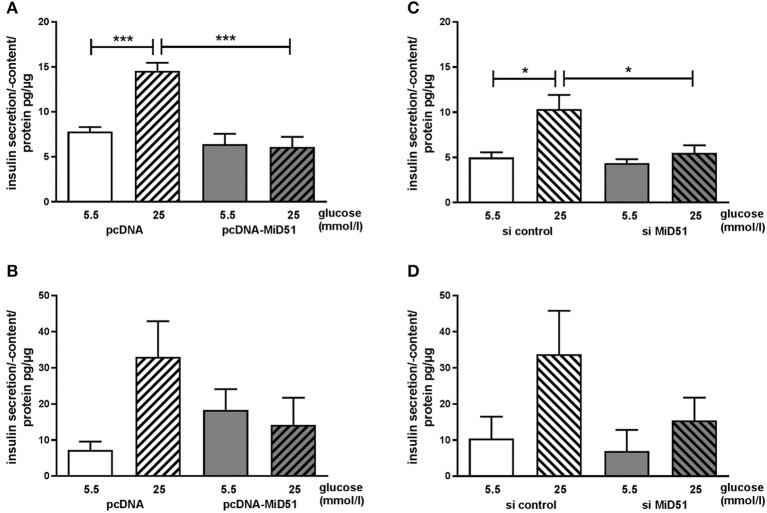
Reduced glucose-stimulated insulin secretion after overexpression and downregulation of MiD51 in MIN6 and primary mouse islet cells. Basal (5.5 mmol/l glucose, open bars) and stimulated (25 mmol/l glucose, striped bars) insulin secretion in MIN6 **(A,C)** and primary mouse islet **(B,D)** cells after transfection with pcDNA **(A,B)** or si control **(C,D)** (white bars) or pcDNA-MiD51 **(A,B)** or si MiD51 **(C,D)** (gray bars). Data are expressed as mean ± SEM from six independent experiments; ^*^p < 0.05, ^***^p < 0.001 (ANOVA/Bonferroni's test).

Control transfected MIN6 cells showed a significant 2.0-fold increase in insulin secretion after stimulation with 25 mmol/l glucose compared with 5.5 mmol/l glucose (Figure 7A). Overexpression of MiD51 resulted in a significant loss of glucose-stimulated insulin secretion (Figure 7A). This finding was similar in primary mouse islet cells: after control transfection there was a 4.7-fold increase in insulin secretion after stimulation with 25 mmol/l glucose compared with 5.5 mmol/l glucose (Figure 7B). However, overexpression of MiD51 resulted not only in total glucose unresponsiveness but also in doubling of the basal insulin secretion rate in mouse islet cells (Figure 7B). MIN6 cells transfected with negative control siRNA also exhibited a significant 2.0-fold increase in insulin secretion after stimulation with 25 mmol/l glucose compared with 5.5 mmol/l glucose (Figure 7C). Downregulation of MiD51 resulted in a significant reduction in insulin secretion after stimulation with 25 mmol/l glucose compared with 5.5 mmol/l glucose. A similar finding was observed in primary mouse islet cells: after control transfection there was a 3-fold increase in insulin secretion after stimulation with 25 mmol/l glucose compared with 5.5 mmol/l glucose, and this was reduced to 2-fold after MiD51 downregulation (Figure 7D).

### Effect of MiD51 Expression on Mitophagy, Mitochondrial Function, and Viability

**Figure 8:**
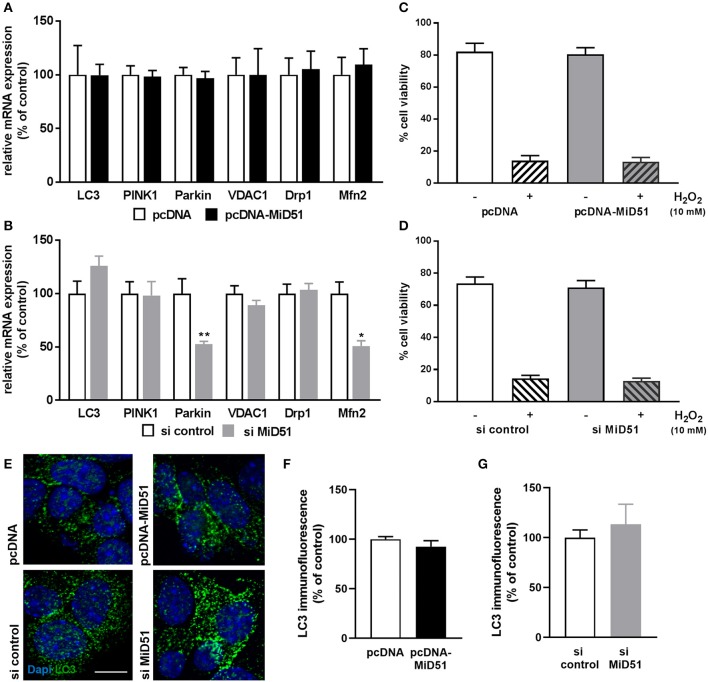
Expression and viability of MIN6 cells. Expression of genes that regulate mitophagy (LC3, PINK1 and Parkin), mitochondrial function (VDAC1) or mitochondrial dynamics (Drp1 and Mfn2) was investigated in MIN6 cells after transfection with **(A)** pcDNA (white bars) or pcDNA-MiD51 (black bars) and **(B)** si control (white bars) or si MiD51 (gray bars). Data are expressed as mean ± SEM from six independent experiments; ^*^p < 0.01, ^**^p < 0.01 (ANOVA/Bonferroni's test). Viability **(C,D)** of MIN6 cells was analyzed using Alamar Blue^®^ after transfection with pcDNA **(C)** or si control **(D)** (white bars) or pcDNA-MiD51 **(C)** or si MiD51 **(D)** (gray bars) and without (open bars) or with (striped bars) 10 mM H_2_O_2_. Data are expressed as mean ± SEM from four independent experiments. MIN6 cells (transfected as indicated) were analyzed by confocal microscopy after immunofluorescence staining with LC3 **(E)**. Representative images from three independent experiments each are shown. Scale bar: 10 μm. At least 15 cells per experiment were automatically analyzed and the mean LC3 expression was calculated. Data are expressed as mean ± SEM in % of controls for overexpression **(F)** and downregulation **(G)**.

Mitophagy is a cellular process of autophagy-based mitochondrial degradation that eliminates dysfunctional mitochondria. To investigate the effect of MiD51 expression level on mitochondrial clearance we measured gene and protein expression of the key autophagy marker LC3 and gene expression of the specific mitochondrial degradation signaling proteins PINK1 and Parkin in transfected MIN6 cells. All remained unchanged after MiD51 overexpression (Figures 8A,E,F). In contrast, Parkin expression was significantly reduced after MiD51 downregulation, whereas no significant changes were observed for LC3 and PINK1 (Figures 8B,E,G). Voltage-dependent anion-selective channel 1 (VDAC1) expression remained stable in response to changes in MiD51 expression (Figures 8A,B). Furthermore, no specific effects on MIN6 cell viability were observed (Figures 8C,D). mRNA expression of the fission protein Drp1 is not altered by either high or low MiD51 expression (Figures 8A,B). The fusion protein Mfn2 was significantly reduced after downregulation of MiD51 (Figure 8B) but remained unchanged after MiD51 overexpression (Figure 8A).

## Discussion

A healthy mitochondrial network is crucial for metabolic homeostasis and insulin secretion in pancreatic beta cells. There is a growing body of evidence to indicate that alterations in mitochondrial metabolism and dynamics lead to beta cell dysfunction (9, 39, 46, 47). A key step in mitochondrial fission is the recruitment of Drp1 to the mitochondrial outer membrane surface by MiD51, among other factors (18, 22–24). We have used MiD51 overexpression and downregulation to investigate the effect of MiD51 on mitochondrial morphology and cellular function in beta cells.

It has been suggested that MiD51 suppresses Drp1 function, resulting in mitochondrial elongation (24, 26, 28). However, other workers have postulated facilitation of Drp1-mediated fission by MiD51 (18, 31). However, because only Drp1 has GTPase activity and can separate mitochondria, MiD51 must act in some way by influencing Drp1. After overexpression of MiD51 we observed clustering of mitochondria to form huge aggregates in MIN6 cells, and it is well-known that mitochondrial cluster formation is evoked by fragmented mitochondria (48). Because MiD51 acts as a receptor for recruitment of cytosolic Drp1, we propose that small mitochondria aggregate at high MiD51 expression. We did not observe tubular elements in the mitochondrial network of MIN6 cells after MiD51 overexpression. The situation was similar in mouse islet cells, where we also found fragmentation and mitochondrial cluster formation. Downregulation of MiD51 in MIN6 cells—and similarly in mouse islet cells—resulted in inhomogeneity of the mitochondrial network, with hyperelongated mitochondria clearly present alongside small fragmented mitochondria. In contrast to the unmodified expression of other fusion and fission proteins at high MiD51 expression, Mfn2—a key regulator of mitochondrial fusion—was significantly reduced at low MiD51 expression. This finding in itself might account for fragmentation, but not for mitochondrial network inhomogeneity and requires further investigation. In summary, MiD51 seems to be important for the organization of the mitochondrial network in beta cells, acting to balance network dynamics rather than as an elongation or fission factor as such. In addition, our results strengthen the hypothesis that the concentration of anchor proteins of Drp1 is precisely regulated in beta cells. We have previously demonstrated that moderate Fis1 expression improves glucose-stimulated insulin secretion, whereas high expression results in loss of glucose responsiveness (45). Furthermore, only small numbers of mitochondrial aggregates were present during moderate Fis1 expression, whereas high Fis1 expression resulted in mitochondrial clustering and formation of loop-shaped mitochondria. Stepwise modulation of MiD51 expression together with Drp1 and also Fis1 and Mff in future studies will be crucial to shed light on this question.

Because of the high demands associated with mitochondrial respiration during insulin secretion, pancreatic beta cells consume large amounts of oxygen. The efficiency with which oxidative phosphorylation is coupled to ATP synthesis is important for insulin secretion. Indeed, overexpression of MiD51 in MIN6 cells reduced the oxygen consumption rate at stimulatory glucose. Consequently, enhanced MiD51 expression also lowered glucose-stimulated insulin secretion in MIN6 cells and mouse islet cells. It is noteworthy here that the maximal oxygen consumption rate and basal insulin secretion remained unaffected in MIN6 cells. This is consistent with data suggesting beta cell dysregulation in consequence of altered mitochondrial dynamics (44, 45). In mouse islet cells we observed higher basal secretion after enhanced MiD51 expression. The data here are limited because first our transfection rate showed high variability and secondly non-beta cells are also affected by this approach, which might have modulating effects on glucagon secretion. Other workers have demonstrated that mitochondrial fragmentation *per se* does not affect insulin secretion (41). However, the degree of mitochondrial network disruption is evidently crucial for the specific conclusions drawn. It has been shown that after downregulation of Drp1 in beta cells the initially homogeneous mitochondrial network becomes highly heterogeneous with elongated, clustered and looped mitochondria. These morphological changes were found to correlate with functional alterations, including mitochondrial membrane potential, ATP generation and a significant loss of glucose-stimulated insulin secretion, whereas basal insulin secretion was also lowered to a lesser extent (44). In the present study downregulation of MiD51 reduced both basal and glucose-stimulated insulin secretion and, in contrast to overexpression, some glucose responsiveness was retained in mouse islet cells. Accordingly, we observed reductions in glucose-mediated and maximal respiration, suggesting impairment of total mitochondrial capacity. Another recently published study likewise showed that MiD51 deficiency impaired mitochondrial respiration in HeLa cells (49).

Mitophagy is an important process for the selective degradation of damaged mitochondria (35, 50). Mitochondrial fission appears to be essential for selective mitophagy because, in order to undergo degradation, dysfunctional mitochondrial material must be separated from the network (36, 37). PINK1 accumulates on the outer membrane of damaged mitochondria and it is usually these mitochondria that undergo fragmentation (51). Finally, binding of Parkin to PINK1 occurs as a consequence of the dissipation of membrane potential and initiates autophagy (35, 50). Although gene expression of PINK1 and Parkin was not induced after overexpression of MiD51 in MIN6 cells, mitochondrial membrane potential in these cells was significantly reduced. We also investigated the expression of the autophagy marker LC3, and in line with our PINK1/Parkin results we found no differences in LC3 expression in MIN6 cells after overexpression of MiD51 compared with control cells. This argues in favor of failed augmentation of mitophagy in MIN6 cells with enhanced MiD51 expression, resulting in mitochondrial clustering. This hypothesis is consistent with very recently published data in HeLa cells, demonstrating that overexpression of MiD51 confers resistance to PINK1-Parkin-dependent mitophagy (49). The same study also provides clear evidence of the converse: MiD51 depletion facilitates PINK1-Parkin-mediated mitophagy at least in part by degradation of Mfn2 (49). After downregulation of MiD51 we found mitochondrial aggregates, reduced mitochondrial membrane potential and reduced Parkin and Mfn2 expression, but only slightly increased LC3 expression. It has been suggested that cells unable to remove clustered mitochondria by mitophagy undergo caspase-mediated apoptosis (48). Furthermore, it has been postulated that loss of MiD51 confers susceptibility to BAX-mediated cell death (49). We did not detect reduced cell viability after overexpression and downregulation of MiD51 in MIN6 cells. This suggests that feedback mechanisms in MIN6 cells differ from those in HeLa cells and fibroblasts, possibly accounting for the absence of mitophagy. There is mounting evidence that autophagy in beta cells is regulated differently: lysosomal degradation of insulin granules suppresses the process (52), thus exerting a definite impact on mitophagy. Future studies of the effect of MiD51 in primary beta cells are needed to test these hypotheses.

In summary, we have shown that MiD51 is expressed in mouse and human pancreatic insulin- positive islet cells. High expression of MiD51 resulted in mitochondrial fragmentation and cluster formation. We postulate abnormally high recruitment of active Drp1 to the outer mitochondrial membrane due to MiD51 accumulation and dimerization (31). It must be borne in mind that cytosolic Drp1 is accessible for different regulatory mechanisms, namely inhibition by ubiquitination and phosphorylation, whereas this is not the case for mitochondrially anchored Drp1 in the oligomeric state (22). Under such conditions, PINK1 cannot bind to the outer mitochondrial membrane and initiate mitophagy, and complexes may develop between fragmented mitochondria, thus supporting mitochondrial cluster formation. Low ATP generation from the resulting mitochondrial network significantly reduces insulin secretion, and the residual aged insulin granules undergo lysosomal degradation (52) that will suppress autophagy and hence mitophagy. Both the overexpression and downregulation of MiD51 further support the hypothesis that regulation of mitochondrial fission plays a key role in maintaining insulin secretion in pancreatic beta cells. Low expression of MiD51 resulted in inhomogeneity of the mitochondrial network in beta cells, mediated at least in part by suppression of Mfn2 and Parkin. To understand the specific feedback loops that adjust the expression of Drp1 anchor proteins on the outer mitochondrial membrane—namely MiD51, Fis1 and Mff—there is a pressing need for continuing work in pancreatic beta cells from healthy subjects and people with type 2 diabetes.

## Data Availability Statement

All datasets generated for this study are included in the article/supplementary material.

## Ethics Statement

All procedures were conducted in accordance with the German Animal Welfare Act 2006 (last amended 2014) and were approved by the State Department of Agriculture, Food Safety and Fisheries, Mecklenburg-Vorpommern (LALLF M-V). Human adult pancreatic islets were donated from biopsies performed during pancreatic surgery, as approved by the ethics committee of University Medicine Greifswald (BB 050/13).

## Author Contributions

JS and SB formulated the study concept, designed and executed experiments, analyzed and interpreted the data, and wrote the manuscript. JW, CW, and RW executed experiments, and analyzed and interpreted the data. All authors approved the final version of the manuscript.

## Conflict of Interest

The authors declare that the research was conducted in the absence of any commercial or financial relationships that could be construed as a potential conflict of interest.

## Abbreviations

Cy5: Cyanine 5 dye
Drp1: Dynamin-related protein 1
FCCP: Carbonyl cyanide 4-(trifluoromethoxy) phenylhydrazone
Fis1: Fission protein 1
GTP: Guanosine triphosphatase
LC3: Light chain 3
MAP1: Microtubule-associated protein 1
Mff: Mitochondrial fission factor
Mfn1: Mitofusin 1
Mfn2: Mitofusin 2
MiD49: Mitochondrial dynamics protein of 49 kDa
MiD51: Mitochondrial dynamics protein of 51 kDa
MIN6: Mouse insulinoma 6
MTDR: MitoTracker^®^ Deep Red FM
OPA1: Optic atrophy protein 1
PINK1: PTEN-induced putative kinase 1
PTEN: Phosphatase and tensin homolog
PVDF: Polyvinylidene fluoride
RIPA: Radioimmunoprecipitation assay
TMRE: Tetramethylrhodamine ethyl ester
VDAC1: Voltage-dependent anion-selective channel.
